# Machine unlearning in brain-inspired neural network paradigms

**DOI:** 10.3389/fnbot.2024.1361577

**Published:** 2024-05-21

**Authors:** Chaoyi Wang, Zuobin Ying, Zijie Pan

**Affiliations:** Faculty of Data Science, City University of Macau, Macao, Macao SAR, China

**Keywords:** machine learning, spiking neural networks, data security, privacy protection, computer vision, brain-inspired ANNs

## Abstract

Machine unlearning, which is crucial for data privacy and regulatory compliance, involves the selective removal of specific information from a machine learning model. This study focuses on implementing machine unlearning in Spiking Neuron Models (SNMs) that closely mimic biological neural network behaviors, aiming to enhance both flexibility and ethical compliance of AI models. We introduce a novel hybrid approach for machine unlearning in SNMs, which combines selective synaptic retraining, synaptic pruning, and adaptive neuron thresholding. This methodology is designed to effectively eliminate targeted information while preserving the overall integrity and performance of the neural network. Extensive experiments were conducted on various computer vision datasets to assess the impact of machine unlearning on critical performance metrics such as accuracy, precision, recall, and ROC AUC. Our findings indicate that the hybrid approach not only maintains but in some cases enhances the neural network's performance post-unlearning. The results confirm the practicality and efficiency of our approach, underscoring its applicability in real-world AI systems.

## 1 Introduction

The advent of Spiking Neuron Models (SNMs) marks a paradigm shift in the field of artificial intelligence, introducing a neural network class that more accurately mirrors human brain functions. Diverging from traditional neural networks that process information continuously, SNMs operate through discrete events or “spikes,” adeptly managing temporal data. This novel approach has propelled significant advancements in fields such as speech recognition, time-series analysis, and neuromorphic computing, thereby solidifying the importance of SNMs in today's technological domain.

Tracing their origins to the early explorations in computational neuroscience, SNMs were developed to encapsulate the dynamic and complex nature of biological neuron activities. These models have progressively evolved from their initial rudimentary forms to intricate systems that precisely emulate neural firing patterns. Their capability to process information akin to biological procedures not only boosts performance in specific tasks but also deepens our comprehension of the neural bases of human cognition and behavior.

Although the learning capabilities of SNMs have been extensively investigated, the concept of machine unlearning within these models is relatively nascent. Traditional AI has focused on accumulating and retaining knowledge, but the increasing importance of data privacy, the right to be forgotten, and adaptability in changing environments have spotlighted the necessity of machine unlearning. This process, entailing the selective deletion of specific information from a model, is vital for adapting to new data, adhering to privacy regulations, and efficiently managing resources.

Present machine unlearning methodologies are predominantly designed for traditional neural networks and involve comprehensive tactics like retraining or data sanitization. However, these methods may not be suitable or efficient for SNMs, given their unique temporal dynamics. This gap highlights the need for research into unlearning strategies that are specifically tailored for SNMs.

This paper aims to bridge this gap in SNM research by delving into the underexplored aspect of machine unlearning. As reliance on SNMs grows in various applications, it becomes crucial to understand how to implement unlearning processes effectively. Our research promises to revolutionize SNM usage, enhancing their adaptability, efficiency, and compliance with evolving data privacy laws.

We propose a novel methodology for selectively erasing learned information in SNMs, focusing on adaptive retraining of synaptic weights and modulation of neuron firing thresholds. Our approach integrates correlation-based neuron selection for retraining with synaptic pruning, aiming to effectively reduce specific learned responses without overhauling the entire neural model. This is supplemented by an adaptive thresholding mechanism to recalibrate neuron sensitivity, aiding targeted unlearning. Our thorough analysis evaluates the impact of these unlearning strategies on key performance metrics such as accuracy, precision, recall, and ROC AUC across various computer vision datasets. The paper provides a detailed evaluation of the trade-offs between unlearning effectiveness and the retention of overall model performance, offering insights into the practical challenges of applying machine unlearning in complex AI architectures.

The contribution in this paper are summarized as follows.

- The research addresses a crucial gap by exploring machine unlearning specifically within SNMs. To the best of our knowledge, this is the first work to address the challenges in implementing machine unlearning in SNMs, and proposes a practical solution.
- This paper introduces innovative strategies for machine unlearning in SNMs, emphasizing selective retraining, synaptic pruning, and adaptive thresholding. These methods enable SNMs to efficiently forget specific information while maintaining network integrity, a significant advancement in adapting SNMs to dynamic data environments and enhancing compliance with data privacy regulations.
- We present experimental validation of the proposed unlearning methods in SNMs, showcasing their efficiency and practical applicability. Our experiments demonstrate major improvements in SNMs' adaptability and responsiveness to changing data, underscoring the techniques' effectiveness in real-world scenarios and marking a substantial advancement in the application of SNMs in dynamic learning environments.

## 2 Related work

### 2.1 Spiking neuron models

SNMs have garnered significant attention in the fields of computational neuroscience and artificial intelligence, thanks to their unique ability to mimic the biological processes of the human brain (Jose et al., 2014; Fang et al., 2021; Wang et al., 2022; Lagani et al., 2023). The foundational theories for SNMs originate from the pioneering work of mathematical descriptions of neural activity (Hua and Smith, 2004). This work was further advanced by researchers like Izhikevich, enhancing our understanding of neural dynamics and spiking behaviors. Studies on neural coding (Auge et al., 2021), have been crucial in demonstrating how neurons encode information through temporal patterns, setting the stage for the development of complex SNM architectures.

The evolution of SNMs has seen a transition from basic integrate-and-fire models to more sophisticated systems like the Spike Response Model (SRM) and Spike-Timing-Dependent Plasticity (STDP) (Ivans et al., 2020; Huyck and Erekpaine, 2022). Researchers like Maass have played a pivotal role in these developments, improving both the biological realism and computational power of SNMs (Bohnstingl et al., 2020). Parallel to these advancements, there has been significant progress in adapting traditional machine learning algorithms to the temporal dynamics of SNMs, paving the way for their application in various domains (Wang et al., 2023; Yan et al., 2023).

SNMs have shown remarkable success in pattern recognition tasks, as demonstrated by the work of Kasabov and others. Their efficiency in processing temporal data has led to breakthroughs in speech and image recognition. The use of SNMs in time-series analysis and neuromorphic computing, explored by researchers like Indiveri, highlights their potential in providing energy-efficient and rapid processing compared to traditional neural networks (Lian et al., 2023; Lv et al., 2023). However, implementing SNMs at scale presents challenges, primarily due to their computational demands (Chen et al., 2023). Recent researches have emphasized the need for optimization in large-scale SNM networks (Lee et al., 2022; Lemaire et al., 2022). This has prompted the development of specialized hardware and software platforms, such as IBM's TrueNorth and Intel's Loihi, which are tailored to efficiently simulate the complex dynamics of SNMs.

### 2.2 Machine unlearning

Machine unlearning, a relatively new concept in the field of artificial intelligence, is garnering increasing attention due to its potential to address data privacy concerns and the need for adaptable AI systems (Wang et al., 2019; Hu et al., 2022; Chundawat et al., 2023; Liu et al., 2023; Pan et al., 2023; Qiu et al., 2023; Xu et al., 2024). This section reviews key research contributions that have shaped our understanding and approaches to machine unlearning.

The foundational concept of machine unlearning is rooted in the broader context of data privacy and the right to be forgotten, as articulated in legislative frameworks like the GDPR. Early researches in this area introduced the basic principles of machine unlearning, highlighting its significance in the era of big data and privacy concerns (Chen et al., 2021; Qu et al., 2023).

One of the pioneering studies in machine unlearning was conducted by Bourtoule et al. (2021), who introduced the concept of “machine unlearning” as a process to efficiently remove specific data from a model's training set. Their work demonstrated that it is possible for machine learning models to forget data in a way that is verifiable and complies with privacy regulations. Research has also focused on the technical challenges of implementing machine unlearning, particularly in complex models (Cao et al., 2018; Zhou et al., 2022). Ginart et al. (2019) delved into the difficulties of unlearning in large-scale machine learning models and proposed methodologies to quantify the effectiveness of unlearning. Their work laid the groundwork for assessing the impact of unlearning processes on model performance and integrity. Another significant contribution is exploring machine unlearning in the context of deep learning, especially in dynamic neural networks (Ma et al., 2023). Their research addressed the challenges of unlearning in neural networks, which are known for their “black box” nature and complex, layered structures. They proposed methods for selective data removal that maintains the overall stability and accuracy of deep learning models (Golatkar et al., 2020; Graves et al., 2021).

Further, studies have explored the practical applications of machine unlearning in various domains. For instance, a number of researches examined the application of machine unlearning in healthcare data analytics, considering the sensitive nature of medical data and the need for compliance with privacy laws (Ullah et al., 2021). In addition, machine unlearning has also been studied in the context of federated learning environments (Golatkar et al., 2023). Researchers have investigated the implementation of unlearning in decentralized data settings, where data privacy and the ability to forget information are crucial (Pan et al., 2023; Zhang et al., 2023).

## 3 Preliminaries

In this section we briefly introduce how SNMs work on a high level. SNMs represent a significant advancement in neural networks, aiming to more closely emulate the behavior of biological neurons. Unlike traditional artificial neurons, SNMs use discrete events or “spikes” to communicate, modeling neural systems in a way that aligns with biological brain processes. These spikes occur when a neuron's membrane potential exceeds a certain threshold, leading to rapid depolarization and repolarization, a process that SNMs seek to replicate computationally.

Each neuron in an SNM is characterized by its membrane potential, *v*(*t*), which changes over time. The dynamics of the membrane potential are described by:


(1) $$\begin{array}{l} \frac{dv\left(t\right)}{dt}=-\frac{1}{\tau_{m}}v\left(t\right)+I\left(t\right) \end{array}$$

Here, τ_*m*_ is the membrane time constant, and *I*(*t*) represents the synaptic input to the neuron. Spike generation in SNMs occurs when the membrane potential *v*(*t*) exceeds a threshold *v*_thresh_:


(2) $$\begin{array}{l} \text{if}v\left(t\right)\geq v_{\text{thresh}}\text{, then a spike is generated} \end{array}$$

After a spike, the neuron's membrane potential is reset to *v*_reset_:


(3) $$\begin{array}{l} v\left(t\right)\leftarrow v_{\text{reset}} \end{array}$$

Synaptic transmission in SNMs involves the interaction between neurons through synapses. The influence of a synaptic input on a neuron's membrane potential is:


(4) $$\begin{array}{l} I_{\text{syn}}\left(t\right)=\underset{j}{\sum }W_{ij}S_{j}\left(t\right) \end{array}$$

where, *W*_*ij*_ denotes the synaptic weight from neuron *j* to neuron *i*, and *S*_*j*_(*t*) is neuron *j*'s spike train.

Spike-Timing-Dependent Plasticity (STDP) is a key learning mechanism in SNMs, adjusting synaptic weights based on spike timing:


(5) $$\Delta W_{ij}=\{\begin{array}{ll} A_{\text{pos}}e^{-\Delta t/\tau_{\text{pos}}} & \text{if }\Delta t>0\\ -A_{\text{neg}}e^{\Delta t/\tau_{\text{neg}}} & \text{if }\Delta t<0 \end{array}$$

where Δ*W*_*ij*_ represents the change in synaptic weight, Δ*t* is the time difference between spikes, and *A*_pos_, *A*_neg_, τ_pos_, and τ_neg_ are parameters of the STDP rule. In SNMs, the integration of neuronal dynamics, spike generation, synaptic transmission, and learning rules like STDP leads to complex network behaviors, which is similar to biological neural networks.

## 4 Methodology

In this section we present the unlearning methodology for SNMs, which contains three phases, namely Selective Retraining, Synaptic Pruning, and Adaptive Thresholding. In particular, Selective Retraining targets and adjusts specific neurons and synapses responsible for learned information that needs to be forgotten, ensuring minimal disruption to the overall network. Synaptic Pruning goes a step further by selectively weakening or eliminating synaptic connections that have been strengthened by the unwanted data, effectively erasing its trace. Adaptive Thresholding complements these methods by dynamically modifying the firing thresholds of neurons based on their interaction with the unlearned data, fine-tuning the network's overall response. Together, these strategies enable SNMs to efficiently and precisely forget specific information, crucial for adapting to changing data environments and maintaining compliance with data privacy standards.

### 4.1 Selective retraining

Selective retraining in Spiking Neuron Models (SNMs) represents a nuanced method for machine unlearning, focusing on retraining specific segments of the network to selectively forget certain data. This technique is pivotal for ensuring that unlearning is precisely targeted, efficient, and minimally disruptive to the overall learned behaviors of the network.

The initial step in this process is to pinpoint the neurons and synapses that were instrumental in learning the specific data intended for unlearning. This identification is conducted by analyzing the spike trains and synaptic modifications incurred during the learning phase. For instance, the correlation of the spike train of neuron *i*, denoted as *S*_*i*_(*t*), with the targeted data *d* for unlearning is calculated through the integral:


(6) $$\begin{array}{l} Corr_{i}\left(d\right)=\int S_{i}\left(t\right)\cdot d\left(t\right)dt \end{array}$$

Neurons showing a high correlation with the data *d* are earmarked for retraining. In parallel, synaptic weights that have significantly altered due to learning *d* are identified. Representing the synaptic weight from neuron *i* to neuron *j* as *W*_*ij*_, and the weight change attributed to *d* as Δ*W*_*ij*_(*d*), synapses are selected based on the extent of this change:


(7) $$\begin{array}{l} \Delta W_{ij}\left(d\right)=W_{ij}^{post}-W_{ij}^{pre} \end{array}$$

Substantial values of Δ*W*_*ij*_(*d*) signify a noteworthy contribution to learning *d*.

Once the key neurons and synapses are identified, retraining involves adjusting these elements using a modified learning rule. For SNMs employing Spike-Timing-Dependent Plasticity (STDP), the standard synaptic weight update rule is:


(8) $$\begin{array}{l} W_{ij}^{new}=W_{ij}+\eta \cdot STDP\left(\Delta t_{ij}\right) \end{array}$$

where η symbolizes the learning rate. In selective retraining, this rule is adapted to:


(9) $$\begin{array}{l} W_{ij}^{\prime }=W_{ij}-\alpha \cdot \Delta W_{ij}\left(d\right)\cdot \kappa_{\left\{i,j\in Selected\right\}} \end{array}$$

Here, $W_{ij}^{\prime }$ represents the revised synaptic weight, α is a retraining factor, and κ_{*i, j*∈*Selected*}_ is an indicator function that activates (1) when both neurons *i* and *j* are within the retraining selection. The retraining factor α is a pivotal element that dictates the degree of synaptic adjustment, calibrated according to the required unlearning level.

Following the retraining phase, the network's performance is evaluated to confirm the effective erasure of data *d*. This evaluation typically includes assessing the network's reaction to *d* and juxtaposing it with the anticipated outcome after unlearning. Should the network's response to *d* closely mirror its state prior to unlearning, additional retraining iterations may be necessitated.

### 4.2 Synaptic pruning

The “Synaptic Pruning” phase in the context of machine unlearning in SNMs is a critical process that involves selectively deactivating or removing synaptic connections that were strengthened during the learning of specific data. This approach aims to erase the neural traces of the data to be unlearned, contributing significantly to the overall machine unlearning process.

The first step in synaptic pruning is to evaluate the contribution of each synapse to the learning of the specific data. This involves analyzing how synaptic strengths have changed in response to the data. If *W*_*ij*_ represents the weight of the synapse between neuron *i* and neuron *j*, and Δ*W*_*ij*_ represents the change in synaptic weight due to learning, the contribution of each synapse can be quantified. The change in weight is calculated as:


(10) $$\begin{array}{l} \Delta W_{ij}=W_{ij}^{post}-W_{ij}^{pre} \end{array}$$

Here, $W_{ij}^{post}$ and $W_{ij}^{pre}$ are the synaptic weights after and before the learning of the specific data, respectively. A high value of |Δ*W*_*ij*_| indicates a significant contribution to the learning process.

Synaptic pruning is performed based on certain criteria, typically involving the magnitude of synaptic change and its relevance to the unlearning process. Synapses are selected for pruning if their change exceeds a predefined threshold, θ, which is determined based on the unlearning requirements. The pruning condition can be mathematically defined as:


(11) $$Prune_{ij}=\{\begin{array}{ll} 1 & \text{if }|\Delta W_{ij}|>\theta \\ 0 & \text{otherwise} \end{array}$$

In this formulation, *Prune*_*ij*_ is a binary variable that indicates whether the synapse between neurons *i* and *j* should be pruned.

Once the synapses to be pruned are identified, the synaptic weights are adjusted accordingly. The pruning process can be mathematically represented as:


(12) $$\begin{array}{l} W_{ij}^{\prime }=W_{ij}\cdot \left(1-Prune_{ij}\right) \end{array}$$

Here, $W_{ij}^{\prime }$ is the new synaptic weight after pruning. If a synapse is selected for pruning (*Prune*_*ij*_ = 1), its weight is set to zero, effectively removing its influence on the network.

The impact of synaptic pruning on the overall functionality of the SNM is carefully evaluated. This involves assessing the network's performance and behavior post-pruning to ensure that only the targeted data has been unlearned and that the network's ability to process other data remains intact. The network's response to various inputs is tested, and if necessary, minor adjustments are made to the pruned synapses to fine-tune the network's performance.

Synaptic pruning in the context of SNMs is a nuanced and critical process that plays a pivotal role in the machine unlearning mechanism. By selectively weakening or removing synapses that contribute to the learning of specific data, synaptic pruning effectively erases the neural representation of that data, aiding in the overall goal of machine unlearning while preserving the integrity and functionality of the network.

### 4.3 Adaptive thresholding

Adaptive thresholding in the context of machine unlearning in SNMs is a sophisticated mechanism for adjusting neurons' firing thresholds. This adjustment is based on their activity in relation to the data targeted for unlearning, ensuring specificity and effectiveness in the unlearning process.

The principle of adaptive thresholding involves modifying the firing threshold of a neuron to reduce its response to stimuli linked with the data to be unlearned. The threshold adjustment for neuron *i* is determined by its response to the unlearning stimulus. The change in the firing threshold, ΔΘ_*i*_, is formulated as:


(13) $$\Delta \Theta_{i}=-\gamma \sum_{t}(S_{i}(t)-{\hat{S}}_{i}(t))$$

In this equation, *S*_*i*_(*t*) represents the actual spike response at time *t*, Ŝ_*i*_(*t*) is the desired response (often reduced or null in response to the unlearned data), and γ is a scaling factor for the threshold adjustment.

The iterative update of the neuron's threshold is defined as:


(14) $$\begin{array}{l} \Theta_{i}^{new}=\Theta_{i}+\Delta \Theta_{i} \end{array}$$

Here, $\Theta_{i}^{new}$ becomes the updated threshold after each iteration, adjusted until the neuron's response aligns with the desired unlearning effect.

To enhance the precision of adaptive thresholding, the spike-timing of the neuron can be incorporated into the threshold adjustment. This involves considering the temporal pattern of spikes in relation to the unlearning data. The revised threshold adjustment can include a term for spike-timing, defined as:


(15) $$\begin{array}{l} \Delta \Theta_{i}^{timing}=\beta \underset{t}{\sum }\left(\frac{dS_{i}\left(t\right)}{dt}-\frac{d{Ŝ}_{i}\left(t\right)}{dt}\right), \end{array}$$

where β is an additional scaling factor and $\frac{dS_{i}\left(t\right)}{dt}$ represents the rate of change of the neuron's spike response.

After updating the firing thresholds, a comprehensive evaluation of the neuron's response to various inputs is conducted. This is to ensure the unlearning process is confined to the targeted data and does not impair the neuron's functionality in processing other data. In addition to individual neuron adjustments, the collective impact on the network is assessed. Network-wide simulations are essential to ensure that the adaptive thresholding of individual neurons does not lead to unintended global changes in network behavior.

### 4.4 Unlearning process

Algorithm 1 describes the whole unlearning process of our proposal. In the first step, neurons and synapses are evaluated for retraining based on the magnitude of their correlation and weight changes, respectively, against set thresholds. Weights are then adjusted according to the learning rate. The second step involves pruning synapses with non-positive contributions to the model. Finally, the third step adapts the firing thresholds of neurons by considering the variance from average spike functions, ensuring the unlearning process is tailored to the dynamics of the SNM.

**Algorithm 1:**
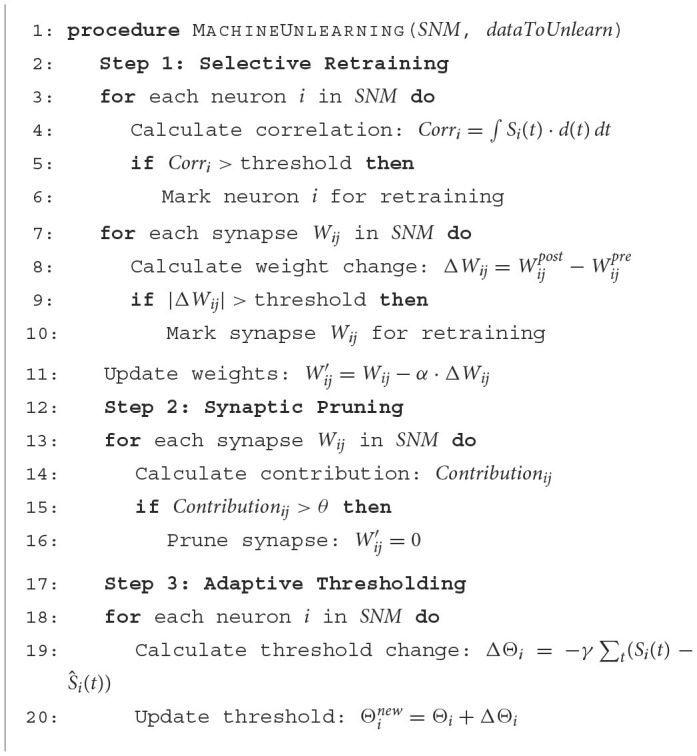
Machine unlearning in spiking neuron models.

## 5 Experiment

In this section we present our empirical evaluation results of our proposal.

### 5.1 Experiment setup

#### 5.1.1 Dataset

We use UCI Human Activity Recognition Dataset (UCI HAR) and MNIST in our experiments. The UCI Human Activity Recognition Dataset comprised sensor data collected from wearable devices, capturing various human activities like walking, sitting, standing, and running. The data included readings from accelerometers and gyroscopes, providing multidimensional time-series data. Each data point in the dataset represented sensor readings at a particular time instance, making it ideal for evaluating the SNMs' capability to process and unlearn temporal patterns. The MNIST consists of grayscale images of handwritten digits (0 to 9). Each image in the dataset is 28 × 28 pixels, and the task involves classifying these images into the correct digit category. The simplicity yet variability of the data make it suitable for testing the SNMs' unlearning ability in a visual pattern recognition context.

#### 5.1.2 Network architecture

The network included two hidden layers, each consisting of 200 neurons. These layers were crucial for capturing the non-linear relationships in the data. The neurons in these layers were connected in a sparse manner, with a connectivity probability of around 0.1, to emulate the sparse connectivity observed in biological neural networks. The output layer's neuron count was determined by the specific requirements of the task, such as the number of classes in a classification task. In our setup, this typically ranged from 10 to 20 neurons. We employed the Izhikevich neuron model across all layers. This model is known for its ability to produce rich firing patterns similar to those observed in real neurons. The STDP rule was employed to adjust synaptic weights based on the relative timing of pre- and post-synaptic spikes.

#### 5.1.3 Training methodology

In our experimental setup, the SNMs were initially trained using a meticulous methodology to ensure robust learning. Synaptic weights were initialized following a Gaussian distribution with a mean of 0 and a standard deviation of 0.05, setting the stage for diverse learning pathways. Training was conducted in batches, each containing 100 data points, utilizing the STDP rule with a learning rate of 0.01 for synaptic adjustments. Homeostatic mechanisms were applied to maintain network stability, including synaptic normalization where weights were scaled to a norm of 1.0 after each training epoch. Dropout with a rate of 20% was employed to prevent overfitting. Cross-validation, using a split of 70% training, 15% validation, and 15% test data, allowed for effective hyperparameter tuning and performance assessment. This approach, integrating biologically inspired learning rules and modern training techniques, was pivotal in achieving a high degree of learning efficiency in the SNMs before applying the unlearning strategies.

### 5.2 Results

In this subsection, we unlearn a special portion of samples in the dataset. We first introduce the evaluation metrics we used to evaluate the unlearned model.

- **Accuracy:** Measures the proportion of correctly classified instances out of the total instances. It's a fundamental metric for assessing the overall effectiveness of the model.
- **Precision:** Indicates the proportion of correctly identified positive instances out of all instances that were predicted as positive.
- **Recall:** Also known as sensitivity, recall measures the proportion of actual positive instances that were correctly identified.
- F1-Score: Represents the harmonic mean of precision and recall. It provides a balance between these two metrics, offering a single measure of the model's performance in scenarios where both false positives and false negatives are crucial.
- Sensitivity: Similar to recall, it measures the true positive rate. It indicates the model's ability to correctly predict positive instances.
- Specificity: Contrary to sensitivity, specificity measures the true negative rate.
- ROC AUC (Receiver Operating Characteristic Area Under Curve): This metric provides an aggregate measure of performance across all possible classification thresholds. It evaluates the trade-offs between true positive rate (sensitivity) and false positive rate (1-specificity) across different thresholds. A higher ROC AUC indicates a better model performance.

We conduct two types of unlearning, namely Class-wise Unlearning and Sample-wise Unlearning. For Class-wise Unlearning, we unlearn a radom class of sample in the whole training set. For Sample-wise Unlearning, we unlearning 10% of samples that uniformly distributed to the original dataset. Tables 1–4 reports the sample-wise unlearning and class-wise unlearning for UCI HAR and MNIST, respectively.

**Table 1:** Sample unlearning performance for UCI HAR.

**Metric**	**Baseline**	**Post-unlearning**	**Post-retraining**
Accuracy	0.855	0.789	0.807
Precision	0.872	0.796	0.809
Recall	0.860	0.738	0.802
F1-score	0.854	0.779	0.883
Sensitivity	0.842	0.753	0.878
Specificity	0.865	0.757	0.887
ROC AUC	0.844	0.793	0.898

**Table 2:** Sample unlearning performance for MNIST.

**Metric**	**Baseline**	**Post-unlearning**	**Post-retraining**
Accuracy	0.949	0.939	0.906
Precision	0.964	0.946	0.908
Recall	0.954	0.888	0.902
F1-score	0.949	0.929	0.975
Sensitivity	0.938	0.903	0.970
Specificity	0.958	0.907	0.978
ROC AUC	0.939	0.943	0.988

**Table 3:** Class unlearning performance for MNIST.

**Metric**	**Baseline**	**Post-unlearning**	**Post-retraining**
Accuracy	0.852	0.827	0.776
Precision	0.887	0.796	0.769
Recall	0.827	0.722	0.773
F1-score	0.886	0.797	0.850
Sensitivity	0.871	0.750	0.907
Specificity	0.856	0.720	0.928
ROC AUC	0.863	0.807	0.856

**Table 4:** Class unlearning performance for UCI HAR.

**Metric**	**Baseline**	**Post-unlearning**	**Post-retraining**
Accuracy	0.691	0.748	0.649
Precision	0.719	0.720	0.643
Recall	0.671	0.653	0.646
F1-score	0.719	0.721	0.711
Sensitivity	0.706	0.678	0.759
Specificity	0.694	0.651	0.776
ROC AUC	0.700	0.730	0.716

Drawing upon the observed data from the four tables presented, we can conclude that machine unlearning has a tangible impact on model performance across different datasets, with each exhibiting a unique pattern of performance degradation and recovery through retraining.

For the UCI HAR dataset, the unlearning process led to a substantial drop in all performance metrics, demonstrating the effectiveness of the unlearning protocol in diminishing the model's accuracy, precision, and other key metrics. Despite this, the retraining process was able to recuperate a considerable portion of the performance, although not entirely to baseline levels.

In contrast, the MNIST dataset displayed a more robust retention of performance post-unlearning, with a less dramatic reduction in metrics. This indicates a potential dataset-specific resilience to the unlearning process. Upon retraining, the MNIST dataset showed a remarkable recovery, with metrics such as the ROC AUC nearly returning to baseline, highlighting the model's ability to relearn effectively after unlearning. Additionally, the class unlearning performance for MNIST reveals a similar trend where unlearning affects the metrics significantly, yet retraining assists in regaining much of the lost performance, albeit with some loss still evident when compared to the original baseline.

**Figure 1:**
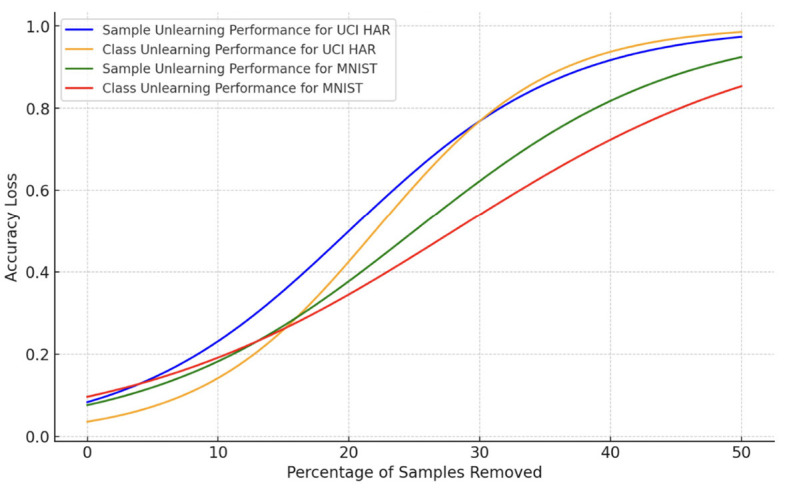
Trade-off between accuracy loss and percentage of samples unlearned.

Next, we report the trade-off between accuracy loss and percentage of samples unlearned in Figure 1. The accuracy loss increases with the percentage of samples removed, which is an expected outcome in machine unlearning scenarios. The UCI HAR dataset, both for sample and class unlearning, shows a slightly lower rate of accuracy decline compared to the MNIST dataset, indicating that it may be more resilient to data removal or that the unlearning process is more effective for this dataset.

We also report the Membership Inference Attack (MIA) accuracy of the unlearned SNM model. Specifically, we infer the unlearned data, to examine if the model still remember the unlearned dataset. The results is reported in Tables 5, 6.

**Table 5:** MIA performance for MNIST dataset.

**Setting**	**Accuracy**	**Precision**	**Recall**
Initial sample performance	90%	89%	88%
Post-unlearning sample performance	88%	87%	86%
Initial class performance	87%	86%	85%
Post-unlearning class performance	85%	84%	83%

**Table 6:** MIA performance for UCI HAR dataset.

**Setting**	**Accuracy**	**Precision**	**Recall**
Initial sample performance	95%	94%	93%
Post-unlearning sample performance	92%	91%	90%
Initial class performance	93%	92%	91%
Post-unlearning class performance	89%	88%	87%

The result show imply that, both the MNIST and UCI HAR datasets have a consistent decline in accuracy, precision, and recall post-unlearning. The MNIST dataset exhibits a slight performance drop, whereas the UCI HAR dataset, despite starting with higher initial performance metrics, follows a similar downward trend. This underscores a universal trade-off between data privacy and model performance across different datasets when applying unlearning techniques.

## 6 Conclusion

This paper represents a significant progression in the field of machine unlearning within biologically inspired neural networks. Our work demonstrates the practicality of implementing machine unlearning in Spiking Neuron Models (SNMs) using a novel approach that combines selective synaptic retraining, pruning, and adaptive thresholding. Through extensive experimentation on diverse datasets, we've shown that while unlearning affects performance metrics like accuracy, precision, and recall, these impacts can be substantially mitigated through strategic retraining and threshold adjustments. This research not only deepens the theoretical understanding of unlearning in complex neural architectures but also offers a practical framework for real-world applications, particularly in addressing data privacy and regulatory compliance, setting the stage for future advancements in neural network maintenance and adaptability.

## Data availability statement

The original contributions presented in the study are included in the article/supplementary material, further inquiries can be directed to the corresponding authors.

## Author contributions

CW: Writing – review & editing, Writing – original draft, Visualization, Validation, Supervision, Software, Resources, Project administration, Methodology, Investigation, Funding acquisition, Formal analysis, Data curation, Conceptualization. ZY: Conceptualization, Data curation, Formal analysis, Funding acquisition, Investigation, Methodology, Project administration, Resources, Software, Supervision, Validation, Visualization, Writing – original draft, Writing – review & editing. ZP: Conceptualization, Data curation, Formal analysis, Funding acquisition, Investigation, Methodology, Project administration, Resources, Software, Supervision, Validation, Visualization, Writing – original draft, Writing – review & editing.
